# Comment on: “Comparison of automatic versus constant CPAP in elderly patients after major abdominal surgery: a randomized noninferiority trial”

**DOI:** 10.1016/j.bjane.2026.844744

**Published:** 2026-03-05

**Authors:** Raghuraman M. Sethuraman, Annu Theagrajan, Akash Babu

**Affiliations:** Sree Balaji Medical College and Hospital, Department of Anesthesiology, Chennai, India

Dear Editor,

We read the research article on the comparison of the automatic versus constant Continuous Positive Airway Pressure (CPAP) in elderly patients after major abdominal surgery with great interest.^1^ We commend the authors for the wonderful concept of comparing these two techniques. However, the trial design and the interpretation of the results warrant further discussion.

First, Thu et al.^1^ published the study as a noninferiority type. Nevertheless, we would like to highlight that a few key elements required to interpret a noninferiority trial were not clearly reported in accordance with the protocol specified for a noninferiority study. Primarily, this type of study should be planned before commencing the study, with calculation of the noninferiority margin (Δ) and the sample size, and these details should be incorporated in the trial registry.^2^^,^^3^ Thu et al.^1^ did not mention the study as “noninferiority” while explaining the study arms in the trial registry, and the Δ-value was also not provided.

Second, the sample size calculation raises an additional concern. Thu et al.^1^ did not mention the calculation of the noninferiority margin. This is pivotal for the sample size calculation.^2^^,^^3^ Consequently, we believe that the sample size calculation may be inaccurate.

Third, reporting of a noninferiority study should ideally include a figure illustrating the position of the confidence interval with the null value and the noninferiority margin.^2^ This was not provided in the current study; hence, the results need careful interpretation, especially considering the p-value was significantly lower for the oxygenation and FVC, suggesting a superiority of the constant CPAP group, rather than “non-inferior”. We would like to seek clarification from the authors whether the primary outcome was “oxygenation”.

In summary, there are many important concepts and protocols to conduct and report a noninferiority study.^2^^,^^3^ Unfortunately, a few key elements, such as the prespecified primary endpoint, the calculation of noninferiority margin (Δ) and its justification, the position of the confidence interval in relation to Δ, were not presented in the current study as discussed above. As the interpretation of the results, consequently the conclusions, are mainly based on the specific protocols of a noninferiority study,^2^^,^^3^ we hope that further insights from Thu et al.^1^ would clarify the readers on these aspects.

## Authors' contributions

RMS: Conceptualization, drafting, reviewing and editing the manuscript. AT, AB: Drafting the manuscript. All authors approved the final version.

## Data availability statement

No new data were created or analyzed in this study. Data sharing is not applicable to this article.

## Declaration of competing interest

The authors declare no conflicts of interest.

## References

[1] Thu N.D., Thuy N.T., Nguyen L.S., Thang C.Q., Thach N.N., Kien NT. (2025). Comparison of automatic versus constant CPAP in elderly patients after major abdominal surgery: A randomized noninferiority trial. Braz J Anesthesiol (English Edition).

[2] Piaggio G., Elbourne D.R., Pocock S.J., Evans S.J.W., Altman D.G., CONSORT Group FT (2012). Reporting of Noninferiority and Equivalence Randomized Trials: Extension of the CONSORT 2010 Statement. JAMA..

[3] Althunian T.A., de Boer A., Groenwold R.H.H., Klungel OH. (2017). Defining the noninferiority margin and analysing noninferiority: An overview. Br J Clin Pharmacol.

